# Assessment of the association of baseline anti-CarbV and anti-MCV antibodies with response to treatment and radiographic progression in an RA population treated with either methotrexate or baricitinib: post-hoc analyses from RA-BEGIN

**DOI:** 10.1186/s13075-020-02284-y

**Published:** 2020-08-18

**Authors:** Pedro López-Romero, Lorena Martinez-Gamboa, Holger Bang, Inmaculada de la Torre, Thorsten Holzkämper, Eugen Feist

**Affiliations:** 1 Eli Lilly & Company, Lilly Corporate Center, Indianapolis, IN USA; 2 Eli Lilly & Company, Avenida de la Industria, 30., 28108 Alcobendas, Madrid Spain; 3 Department of Rheumatology and Clinical Immunology, Charité – Universitätsmedizin, Charitéplatz, 10117 Berlin, Germany; 4 ORGENTEC Diagnostika GmbH, Carl-Zeiss-Straße 49, 55129 Mainz, Germany; 5 Helios Department of Rheumatology, Sophie-v.-Boetticher-Straße 1, 39245 Gommern, Germany

**Keywords:** Rheumatoid arthritis, Baricitinib, Autoantibodies, Anti-CarbV

## Abstract

**Background:**

The development of autoantibodies in patients with rheumatoid arthritis (RA) has potential as a marker of treatment response. This analysis assessed the association of an autoantibody response to carbamylated vimentin (anti-CarbV) and to vimentin modified by citrullination (anti-MCV) with response to treatment and structural damage progression in the phase III study RA-BEGIN.

**Methods:**

Data from patients in the modified intent-to-treat population of RA-BEGIN were included for analysis; these patients received methotrexate (MTX), baricitinib 4 mg once daily, or baricitinib plus MTX during the 52-week study period. Endpoints analyzed were clinical response to treatment, assessed using change from baseline (CFB) in Simplified Disease Activity Index (SDAI) and Disease Activity Score for 28-joint count with serum high-sensitivity C-reactive protein (DAS28-hsCRP), and structural damage progression, assessed using CFB greater than the smallest detectable change in the van der Heijde-modified Total Sharp Score. The anti-CarbV and anti-MCV isotypes assessed were immunoglobulin (Ig) A, IgG, and IgM. Multivariable mixed-effect models for repeated measures (MMRMs) were used for the longitudinal analysis of treatment response, and multivariable logistic regression models were used for the analysis of structural damage progression at week 52.

**Results:**

Analysis of the association between autoantibodies and treatment response showed that high titers of anti-CarbV (IgA and IgG) were associated with a greater clinical response as measured by SDAI and DAS28-hsCRP. Anti-CarbV IgA and IgG, but not IgM, demonstrated an association after adjustment for other factors included in the MMRMs. High titers of anti-CarbV IgM were associated with a poor response to MTX monotherapy, whereas a nonsignificant trend toward a better response to baricitinib and baricitinib plus MTX was observed. There was no association between anti-MCV antibodies and treatment response. High titers of anti-CarbV IgA were associated with a greater probability of radiographic progression, but no association between anti-MCV antibodies and radiographic progression was observed.

**Conclusions:**

High titers of anti-CarbV IgA and IgG isotypes, but not anti-MCV isotypes, may be useful prognostic biomarkers for identifying the likelihood of the response to treatment and structural damage progression in patients with RA.

## Introduction

The Janus kinase/signal transducer and activator of transcription (JAK/STAT) pathway is known to be involved in the pathogenesis of the chronic inflammatory disease rheumatoid arthritis (RA) [1, 2]. Many proinflammatory cytokines signal through JAKs [2]; thus, JAK inhibitors represent an effective treatment option in patients with RA. There are four members of the JAK family: JAK1, JAK2, JAK3, and tyrosine kinase 2 (Tyk2) [1]; baricitinib is an oral selective inhibitor of JAK1 and JAK2, with less effect on JAK3 and Tyk2 [3, 4]. The efficacy and safety of baricitinib were established in four phase III, randomized, double-blind, multicenter studies in patients with active RA [5–8]; in these studies, baricitinib demonstrated good clinical efficacy and clear inhibition of radiographic progression and was superior to methotrexate (MTX) and adalimumab with respect to treatment response [7, 8]. Baricitinib is currently approved in more than 65 countries worldwide, and over 100,000 patients with RA have been treated to date (Eli Lilly & Company, data on file).

Autoantibodies are frequently involved in the pathogenesis of RA. The development of autoantibodies has been linked to a specific genetic background (the so-called shared epitope), and evidence also suggests they can be induced by environmental factors, such as smoking [9]. Often autoantibodies develop before the onset of RA symptoms and can serve as diagnostic markers [10, 11]. Patients with RA can generally be stratified into two subgroups (seropositive/seronegative) based on autoantibody prevalence, usually defined by the presence/absence of rheumatoid factor (RF) and/or anti-citrullinated protein antibody (ACPA) [12–14]; post-translational modification of proteins by citrullination has been linked to RA, and ACPA represents a highly specific marker for this disease [12, 13]. The targeted antigens of ACPA are modified autoantigens such as vimentin, alpha-enolase, and fibrinogen [12, 13, 15]. It has been reported that seropositive patients have a higher risk of severe and systemic disease than seronegative patients [16] and that these antibodies are also associated with certain comorbidities, such as coronary heart disease [17]. Therefore, treatment recommendations for seropositive patients with RA differ from those for seronegative patients, promoting earlier and more aggressive treatment [14].

Recently, an autoimmune response against other post-translational modifications, carbamylation (or homo-citrullination) and acetylation, has also been described in RA [18–21]. Of note, carbamylation has also been linked to environmental factors, such as exposure to cyanate through smoking, and to comorbidities, such as kidney dysfunction [19, 20]. However, the diagnostic performance and clinical relevance of autoantibodies to carbamylated proteins (anti-CarbP) have not been fully clarified, and these antibodies are currently not included among the standard diagnostic markers for RA [14]. However, it is possible that the clinical characteristics or serological markers present in patients with RA with an anti-CarbP response could be used to evaluate the prognosis of these patients and may help to improve treatment.

The objectives of the present analysis were to assess the association of an autoantibody response to carbamylated vimentin (anti-CarbV) and to vimentin modified by citrullination (anti-mutated citrullinated vimentin; anti-MCV) with response to treatment and structural damage progression in the phase III study RA-BEGIN.

## Methods

### RA-BEGIN study design

RA-BEGIN was a phase III, 52-week, double-blind, three-arm, multicenter study assessing the efficacy and safety of oral baricitinib 4 mg once daily as monotherapy or in combination with MTX versus MTX monotherapy in patients with active RA who had no or limited prior treatment with conventional synthetic disease-modifying antirheumatic drugs (DMARDs; a maximum of three previous doses of MTX) and were naïve to biologic DMARDs [7]. MTX was initiated at 10 mg/week and was increased to 20 mg/week by week 8 if well tolerated. Further details of the RA-BEGIN study design can be found in the primary publication [7].

### Endpoints for current analysis

The endpoints of interest for these analyses were clinical response to treatment and structural damage progression. Clinical response to treatment was assessed by the change from baseline (CFB) at weeks 4, 12, 16, 20, 24, 32, 40, and 52 and was analyzed using the Simplified Disease Activity Index (SDAI) score [22] and Disease Activity Score for 28-joint count with serum high-sensitivity C-reactive protein (DAS28-hsCRP) [23, 24]. Structural damage progression was defined for this analysis as the binary variable CFB at week 52 greater than the smallest detectable change (SDC) in the van der Heijde-modified Total Sharp Score (mTSS) [25, 26]; the SDC is defined as the minimum amount of change in a patient’s score that can be assessed beyond measurement error. SDC was calculated according to the method of Bruynesteyn et al. [27]. In RA-BEGIN, the estimated SDC in mTSS at week 52 was 1.4. Additional details on the assessment of radiographs for structural joint damage in RA-BEGIN are provided in the methods section of Additional file 1.

### Determination of antibody reactivity

Baseline serum samples were analyzed for immunoglobulin (Ig) G, IgM, and IgA reactivity toward MCV and CarbV using enzyme-linked immunosorbent assay (ORGENTEC Diagnostika GmbH, Mainz, Germany) [20, 28]. The detection range of each assay used to determine antibody concentrations was 0.1 to 1000. As values of < 0.1 and > 1000 could not be accurately determined by the assay, they were given as < 0.1 and > 1000. For analytical purposes, values < 0.1 were set to 0.1 and values > 1000 were set to 1000 for statistical modeling. Serum samples provided were labeled with pseudonyms. Thus, measurement of antibody concentrations was conducted in a blinded manner.

### Analysis population

Data from patients in the modified intent-to-treat (mITT) population of RA-BEGIN were included for analysis. Patients without baseline antibody data or baseline data for any of the covariates used in the corresponding statistical models for each particular endpoint (SDAI and DAS28-hsCRP) and patients without both baseline and post-baseline radiographic data were excluded from statistical analysis.

### Statistical models

Multivariable mixed-effect models for repeated measures (MMRM) [29] were used for the longitudinal analysis of treatment response (SDAI and DAS28-hsCRP). The MMRM included the following variables as fixed effects: treatment (MTX, baricitinib 4 mg, baricitinib 4 mg + MTX), visit (weeks 4, 12, 16, 20, 24, 32, 40, and 52), baseline antibody of interest (U/mL), baseline SDAI (or DAS28-hsCRP), baseline ACPA (U/mL), baseline RF (U/mL), presence of erosions at baseline (yes/no), age (years), sex, baseline body mass index (BMI; kg/m^2^), geographic region (Europe/Japan/Rest of the World/USA and Canada/Central and South America and Mexico), and treatment-by-visit interaction. Patient was included in the MMRM as a random effect, and a compound symmetry covariance matrix was used to model between- and within-subject errors. This MMRM defined a linear association between the baseline antibody of interest and the response variable (SDAI or DAS28-hsCRP). Additional MMRM, including a natural cubic spline with 3 degrees of freedom to allow nonlinear associations between the baseline antibody serum concentration and the response variable, were fitted. In addition, MMRM including a baseline antibody-by-treatment interaction were fitted to assess whether the association between baseline antibody and response differed according to treatment received at randomization. The MMRM were all nested models, and likelihood-ratio chi-squared tests (LRT) were employed to choose the best competing model.

Multivariable logistic regression (MLR) models were used for the analysis of structural damage progression at week 52. Following a previous MLR used for the analysis of structural progression in RA-BEGIN [30, 31], the association between baseline antibodies and structural progression was estimated using an MLR that included the following factors: treatment, baseline antibody of interest (U/mL), baseline ACPA (U/mL), baseline RF (U/mL), presence of erosions at baseline (yes/no), baseline Clinical Disease Activity Index (CDAI), baseline high-sensitivity C-reactive protein (hsCRP; mg/L), baseline hemoglobin (g/dL), baseline Health Assessment Questionnaire-Disability Index (HAQ-DI), age (years), sex, baseline BMI (kg/m^2^), smoker (yes/no), and geographic region (Europe/Japan/Rest of the World/USA and Canada/Central and South America and Mexico). Adjusted odds ratios (ORs) with corresponding 95% confidence intervals and *p* values were estimated for all factors included in the MLR used to assess associations between baseline anti-CarbV or anti-MCV antibodies and structural progression. As in the MMRM analyses, LRTs were employed for model selection purposes and to assess the type of association between baseline variables and response. Adjusted ORs for baseline antibodies were converted into corresponding adjusted probabilities of structural progression as a function of baseline antibody serum concentrations.

In the MMRM analyses, modified last observation carried forward (mLOCF) was used to handle post-baseline SDAI and DAS28-hsCRP data after the occurrence of intercurrent events, defined as events occurring after randomization and treatment initiation that either precluded observation of the variable or affected its interpretation (e.g., use of rescue medication, treatment discontinuation, loss to follow-up, or death). Similarly, in the MLR analyses, mTSS data at week 52 were imputed using linear extrapolation. Additional details regarding the handling of missing data after the occurrence of intercurrent events can be found in the statistical methods section of Additional file 1.

The multivariable analyses provided estimates of the relative contribution of each factor in the model to the response variable. Therefore, the estimated associations of baseline antibodies with clinical response and structural progression were independent of the effects of other factors included in the models. Natural cubic splines with 3 degrees of freedom were used to model nonlinear associations between the baseline antibody isotype of interest and the response variable (see the statistical methods section of Additional file 1). The estimated coefficients corresponding to the natural cubic splines did not allow for any meaningful clinical interpretation, and effects plots were therefore used to visualize the adjusted means of the response variable as a function of the baseline serum concentration of the different antibodies. Adjusted means for SDAI and DAS28-hsCRP responses and adjusted probabilities for structural progression displayed in the effects plots were estimated from the multivariable models, with continuous covariates fixed at their mean values and categorical covariates fixed at their proportional distribution in the data. A *p* value < 0.05 was considered statistically significant. All statistical analyses were performed using R version 3.5.1 [32].

## Results

### Analysis populations

RA-BEGIN included 584 patients in the mITT population (210 receiving MTX, 159 receiving baricitinib 4 mg/day, and 215 receiving baricitinib 4 mg/day + MTX). Baseline characteristics of the mITT patients have been presented in the primary publication [7]. Of the 584 mITT patients, antibodies were measured in only 570 patients at baseline: 203 receiving MTX, 157 receiving baricitinib 4 mg/day, and 210 receiving baricitinib plus MTX. Of these 570 patients, 431 (75.6%), 521 (91.4%), 446 (78.3%), 149 (26.1%), 557 (97.7%), and 206 (36.1%) were positive for anti-CarbV (IgA, IgG, IgM) and anti-MCV (IgA, IgG, IgM), respectively.

Additionally, and as a consequence of missing data for the variables employed in the models used for analysis of the different endpoints, a total of 550 patients were included in the SDAI analysis (MTX: 192; baricitinib: 155; baricitinib + MTX: 203), 556 patients in the DAS28-hsCRP analysis (MTX: 195; baricitinib: 157; baricitinib + MTX: 204), and 526 patients in the structural damage progression (radiographic) analysis (MTX: 182; baricitinib: 150; baricitinib + MTX: 194).

### Association of baseline anti-CarbV and anti-MCV antibodies with treatment response

**Fig. 1:**
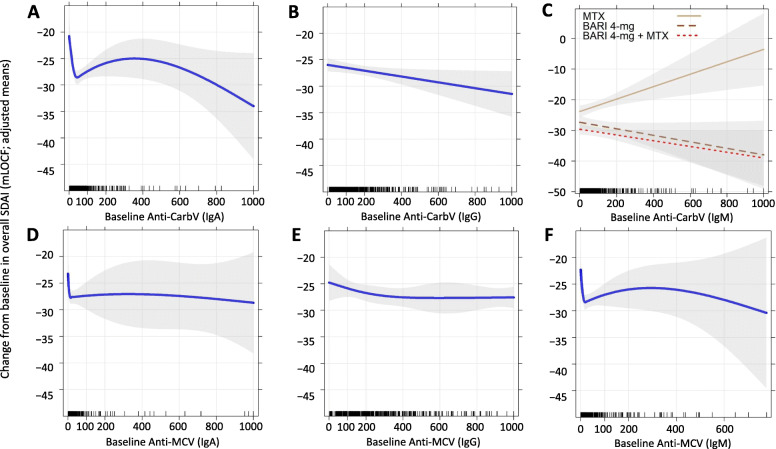
Adjusted means for the association between baseline anti-CarbV/anti-MCV antibodies and CFB in SDAI response. **a** Anti-CarbV (IgA). **b** Anti-CarbV (IgG). **c** Anti-CarbV (IgM). **d** Anti-MCV (IgA). **e** Anti-MCV (IgG). **f** Anti-MCV (IgM). Adjusted ORs for baseline antibodies were converted into corresponding adjusted probabilities of SDAI as a function of baseline antibody serum concentrations. Adjusted overall SDAI means as a function of serum baseline antibody concentrations were estimated using multivariable MMRM with continuous covariates fixed at their mean values and categorical covariates fixed at their proportional distribution in the data. Overall SDAI response was estimated from MMRM, averaging SDAI responses at all post-baseline visits (weeks 4, 12, 16, 20, 24, 32, 40, and 52). *p* values < 0.05 were considered statistically significant. **a** A statistically significant nonlinear association was found for anti-CarbV (IgA). Patients with higher baseline anti-CarbV (IgA) values were more likely to show an overall improvement in SDAI response (*p* = 0.002). **b** A significant linear association was found for anti-CarbV (IgG) (*p* = 0.033). **c** A significant baseline anti-CarbV (IgM)-by-treatment interaction was found (*p* < 0.001). The association between anti-CarbV (IgM) and SDAI depends on the treatment received at baseline. Patients randomized to MTX who had higher baseline anti-CarbV (IgM) were more likely to show lower overall SDAI improvement (*p* = 0.0021). An opposite association was observed for patients randomized to baricitinib; however, neither association was statistically significant (baricitinib, *p* = 0.0874; baricitinib + MTX, *p* = 0.0636). **d**, **e** No statistically significant association for anti-MCV (IgA) [*p* = 0.323] or IgG [*p* = 0.503] was observed. **f** A statistically significant nonlinear association was found for anti-MCV (IgM) (*p* = 0.036). BARI, baricitinib; CarbV, carbamylated vimentin; CFB, change from baseline; Ig, immunoglobulin; MCV, mutated citrullinated vimentin; mLOCF, modified last observation carried forward; MMRM, mixed model for repeated measures; MTX, methotrexate; ORs, odds ratios; SDAI, Simplified Disease Activity Index

*p* values for factors included in the different MMRM used to model the associations of anti-CarbV and anti-MCV antibodies with overall SDAI response are presented in Table 1. The adjusted means for overall SDAI response as a function of baseline anti-CarbV and anti-MCV antibodies estimated using multivariable MMRM are presented in Fig. 1a–f. There was no evidence of baseline antibody-by-treatment interactions for five of the six antibody isotypes analyzed, and the adjusted means for the SDAI response as a function of baseline antibody were similar for the three treatment arms (i.e., the association between antibody and SDAI response was not modified by treatment effect) for these five antibody isotypes. There was a statistically significant baseline antibody-by-treatment interaction for anti-CarbV (IgM) [*p* <  0.001]. In this case, the adjusted means for the SDAI response as a function of the baseline antibody differed for the different treatment arms, as shown in Fig. 1c. The adjusted means presented in Fig. 1 were independent of baseline SDAI and all other factors included in the MMRMs.

**Table 1:** *p* values for MMRM analysis modeling the association of anti-CarbV/anti-MCV antibodies with SDAI response

MMRM variable	Anti-CarbV antibody isotypes			Anti-MCV antibody isotypes		
	IgA	IgG	IgM	IgA	IgG	IgM
Time	< 0.001	< 0.001	< 0.001	< 0.001	< 0.001	< 0.001
Treatment	< 0.001	< 0.001	< 0.001	< 0.001	< 0.001	< 0.001
Baseline antibody (only in MMRM with linear association)	NA	0.033	0.981	NA	NA	NA
Baseline antibody NCS (in MMRM with nonlinear association)	0.002	NA	NA	0.323	0.503	0.036
Baseline SDAI	< 0.001	< 0.001	< 0.001	< 0.001	< 0.001	< 0.001
Baseline ACPA	0.702	0.759	0.957	0.912	0.863	0.980
Baseline RF	0.120	0.123	0.965	0.109	0.155	0.132
Erosions	0.301	0.447	0.375	0.317	0.302	0.318
Age	0.410	0.378	0.430	0.365	0.372	0.168
Sex	0.826	0.753	0.870	0.839	0.749	0.656
BMI	0.763	0.881	0.716	0.830	0.860	0.873
Geographic region	0.020	0.045	0.071	0.056	0.053	0.057
Time-by-treatment interaction	0.633	0.633	0.633	0.633	0.633	0.633
Baseline antibody-by-treatment interaction	NA	NA	< 0.001	NA	NA	NA

Different nested MMRM were compared employing a chi-squared LRT to select between linear (baseline antibody in the MMRM) or nonlinear associations defined with a natural cubic spline (baseline antibody using an NCS in the MMRM) and to test baseline antibody-by-treatment interactions. *p* values < 0.05 were considered statistically significant. The reported *p* values obtained from the multivariable model are for the association between the corresponding factor and overall SDAI response, measured as CFB, while controlling for the influence of all other factors included in the model: SDAI; time (visit: weeks 4, 12, 16, 20, 24, 32, 40, and 52); treatment (MTX, baricitinib 4 mg, baricitinib 4 mg + MTX); baseline antibody, in MMRM with linear association or baseline antibody using NCS, in MMRM with NCS with 3 degrees of freedom (nonlinear association); ACPA (yes/no); RF (yes/no); erosions: presence of erosions at baseline (yes/no); geographic region (Central and South America and Mexico; Europe; Japan; Rest of the World, USA and Canada); BMI; baseline antibody-by-treatment interaction, only in MMRM with (significant) baseline antibody-by-treatment interaction

*ACPA* anti-citrullinated protein antibody, *BMI* body mass index, *CarbV* carbamylated vimentin, *CFB* change from baseline, *Ig* immunoglobulin, *LRT* likelihood-ratio test, *MCV* mutated citrullinated vimentin, *MMRM* mixed model for repeated measures, *MTX* methotrexate, *NA* factor not estimated in the MMRM used for that specific antibody, *NCS* natural cubic spline, *RF* rheumatoid factor, *SDAI* Simplified Disease Activity Index

A statistically significant nonlinear association was found for baseline anti-CarbV (IgA) and SDAI response. Regardless of treatment and other factors included in the MMRM, patients with higher baseline anti-CarbV (IgA) titers were more likely to show an overall improvement in SDAI (*p* = 0.002) (Fig. 1a). Similarly, a statistically significant linear association was found for anti-CarbV (IgG). For the three treatment arms, patients with higher baseline anti-CarbV (IgG) were more likely to show an overall improvement in SDAI (*p* = 0.033) (Fig. 1b). A statistically significant treatment-by-anti-CarbV (IgM) interaction was found for this antibody isotype (*p* <  0.001; Table 1). In this case, the association between baseline anti-CarbV (IgM) and overall SDAI response differed according to the treatment received at baseline; patients who received MTX at baseline who had lower baseline anti-CarbV (IgM) were more likely to show an improvement in SDAI response (*p* = 0.0021) (Fig. 1c). Conversely, patients randomized to either baricitinib or baricitinib plus MTX who had higher anti-CarbV (IgM) showed an improvement in SDAI, but none of these associations were statistically significant for anti-CarbV (IgM) (baricitinib, *p* = 0.0874; baricitinib + MTX, *p* = 0.0636) (Fig. 1c). There was no statistically significant association for anti-MCV (IgA) (*p* = 0.323; Fig. 1d) nor for anti-MCV (IgG) (*p* = 0.503; Fig. 1e).

Finally, a statistically significant nonlinear association with SDAI response was found for anti-MCV (IgM). Patients with higher baseline anti-MCV (IgM) showed a statistically significant improvement in overall SDAI (*p* = 0.036) (Fig. 1f). Similar results were obtained for the association between baseline anti-CarbV or anti-MCV and DAS28-hsCRP, except in patients with higher baseline anti-MCV (IgM), who showed an improvement in overall DAS28-hsCRP that was not statistically significant (*p* = 0.117). Further results are provided in Table S1 and Fig. S1 in Additional file 1.

Overall, no statistical associations between anti-MCV IgA or IgG isotypes and treatment response for SDAI were found. Results for the association between baseline anti-MCV and treatment response for the IgM antibody isotype were inconclusive.

Regardless of the serum concentration of baseline antibodies and other factors included in the MMRM, patients randomized to either baricitinib plus MTX or baricitinib alone showed a statistically significantly greater SDAI and DAS28-hsCRP response at all post-baseline visits. The adjusted means for the CFB in SDAI, estimated in the MMRM used for the analysis of anti-CarbV (IgA), for the three treatment arms and at each post-baseline visit are shown in Fig. S2 in Additional file 1. Despite the significant interaction between anti-CarbV (IgA) and treatment response (Fig. 1c), the improvement in SDAI response was consistently higher and statistically significantly different for baricitinib plus MTX and baricitinib compared with that in patients randomized to MTX. Similar results were obtained for DAS28-hsCRP response (see Fig. S1C in Additional file 1).

Fig. S3 in Additional file 1 shows the adjusted overall SDAI means for the different geographic regions estimated with the MMRM used for the analysis of anti-CarbV (IgA). There was a statistically significant difference in overall SDAI response (averaged over all post-baseline visits) between the USA and Canada compared with Central and South America and Mexico (*d* = 4.538, *p* = 0.001) and between the USA and Canada compared with the Rest of the World (d = 3.218, *p* = 0.045), with a lower overall improvement in SDAI in the USA and Canada in both cases.

### Association of baseline anti-CarbV and anti-MCV antibodies with structural damage progression

**Fig. 2:**
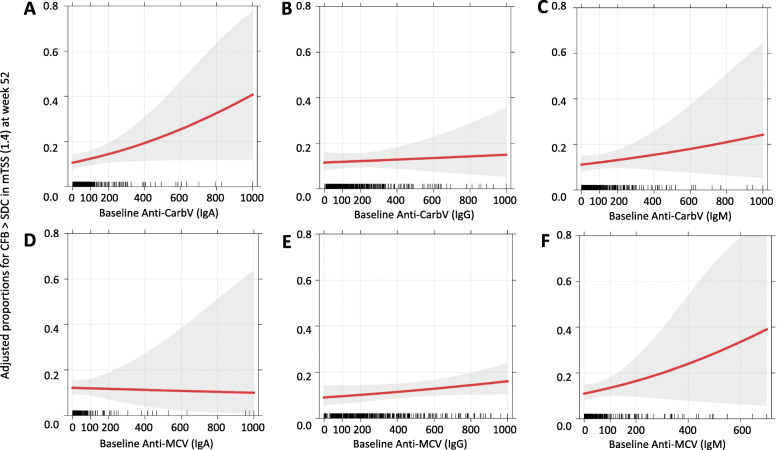
Adjusted probabilities for structural damage progression estimated from MLR for six different baseline antibodies. **a** Anti-CarbV (IgA). **b** Anti-CarbV (IgG). **c** Anti-CarbV (IgM). **d** Anti-MCV (IgA). **e** Anti-MCV (IgG). **f** Anti-MCV (IgM). Adjusted ORs for baseline antibodies were converted into corresponding adjusted probabilities of structural progression as a function of baseline antibody serum concentrations. Adjusted probabilities for structural progression (measured as CFB > SDC [1.4]) for the different baseline antibodies were estimated using MLR with continuous covariates fixed at their mean values and categorical covariates fixed at their proportional distribution in the data. **a** Patients with higher baseline anti-CarbV (IgA) titers were more likely to show structural progression (OR = 1.002), but this association was not statistically significant (*p* = 0.051). **b**, **c** No association between anti-CarbV (IgG) or (IgM) and structural progression was observed (IgG: OR = 1.0; *p* = 0.665; IgM: OR = 1.001; *p* = 0.336). **d**–**f** No association between any anti-MCV isotype and structural progression was observed (IgA: OR = 1.000; *p* = 0.875; IgG: OR = 1.001; *p* = 0.120; IgM: OR = 1.002; *p* = 0.207). CarbV, carbamylated vimentin; CFB, change from baseline; Ig, immunoglobulin; MCV, mutated citrullinated vimentin; MLR, multivariable logistic regression; SDC, smallest detectable change; OR, odds ratio

Adjusted ORs for all factors included in the MLR used to estimate the associations between baseline anti-CarbV and structural progression are presented in Table 2. Patients with higher baseline anti-CarbV (IgA) were more likely to show a larger CFB > SDC (1.4) at week 52 (OR = 1.002), but this association was not statistically significant (*p* = 0.051). An OR of 1.002 can be interpreted as a 0.2% increase in the odds of structural progression when baseline anti-CarbV (IgA) titers are increased by 1 unit and other variables used in the MLR analysis remain fixed (Table 2). The adjusted probability of structural progression as a function of the values of baseline anti-CarbV (IgA) serum concentrations is presented in Fig. 2a. After multivariable adjustment in the MLR analysis of baseline anti-CarbV (IgA), patients randomized to baricitinib plus MTX (OR = 0.307; *p* <  0.001) or to baricitinib (OR = 0.534; *p* = 0.043) were less likely to show structural progression than patients randomized to MTX (Table 2). Also, patients with higher baseline hsCRP levels (OR = 1.017; *p* <  0.001), smokers (OR = 2.045; *p* = 0.026), and patients with lower baseline HAQ-DI scores (OR = 0.614; *p* = 0.034) were more likely to show statistically significant structural progression at week 52. These factors demonstrated a consistent statistical association with structural progression in all the MLR analyses used for the six different antibody isotypes. Additionally, patients with lower BMI were more likely to show structural progression in all MLR analyses except for the analysis of anti-CarbV (IgA). Patients with higher baseline CDAI were also more likely to show structural progression in the multivariable setting, but only in the anti-MCV (IgA) model (see Tables S2–6 in Additional file 1).

**Table 2:** Adjusted ORs from MLR: association between anti-CarbV (IgA) and structural damage progression

MLR variable	OR	Lower CI	Upper CI	*p* value
Baricitinib vs. MTX	0.534	0.287	0.970	0.043
Baricitinib + MTX vs. MTX	0.307	0.159	0.574	< 0.001
Baseline anti-CarbV (IgA)	1.002	1.000	1.004	0.051
Baseline ACPA	1.000	0.999	1.000	0.185
Baseline RF	1.000	0.999	1.001	0.724
Erosions at baseline (yes vs. no)	1.516	0.870	2.715	0.150
Baseline hemoglobin	0.796	0.626	1.009	0.061
Baseline hsCRP	1.017	1.008	1.027	< 0.001
Age	1.000	0.981	1.020	0.969
Sex (male vs. female)	0.614	0.282	1.288	0.207
Baseline BMI	0.948	0.897	1.000	0.051
Smoker (yes vs. no)	2.045	1.084	3.825	0.026
Baseline HAQ-DI	0.614	0.389	0.961	0.034
Baseline CDAI	1.022	1.000	1.047	0.062
Europe vs. CSAM	1.423	0.590	3.325	0.421
Japan vs. CSAM	1.132	0.482	2.653	0.775
Rest of the World vs. CSAM	1.068	0.469	2.390	0.874
USA and Canada vs. CSAM	0.627	0.257	1.436	0.283

Adjusted ORs from the MLR model with corresponding 95% CIs and *p* values. *p* values < 0.05 were considered statistically significant

*ACPA* anti-citrullinated protein antibody, *BMI* body mass index, *CarbV* carbamylated vimentin, *CDAI* Clinical Disease Activity Index, *CI* confidence interval, *CSAM* Central and South America, *HAQ-DI* Health Assessment Questionnaire-Disability Index, *hsCRP* high-sensitivity C-reactive protein, *Ig* immunoglobulin *MLR* multivariable logistic regression, *MTX* methotrexate, *OR* odds ratio, *RF* rheumatoid factor

For all antibody isotypes analyzed, no association between baseline serum concentration and structural progression was observed (Fig. 2a–f). Additionally, there was no statistical evidence of any interaction between these antibodies and treatment (i.e., the associations were not significant and so did not depend on the treatment received at baseline).

## Discussion

JAK inhibitors have become an established treatment option for patients with RA [14]; however, as with other RA treatments, not all patients respond to JAK inhibitor therapy. Since patients can be stratified based on autoantibody prevalence, we evaluated the association between the serum concentration of carbamylated and citrullinated vimentin antibodies and response to treatment and structural progression, in a treatment-naïve patient population who received either MTX, baricitinib, or baricitinib plus MTX in RA-BEGIN [7].

This analysis showed that the association between antibodies and treatment response was different for anti-CarbV or anti-MCV. There was no association between ACPA-related anti-MCV antibodies and treatment response; however, high titers of anti-CarbV (IgA and IgG) were associated with a greater clinical response as measured by SDAI and DAS28-hsCRP. Interestingly, IgA and IgG but not IgM anti-CarbV demonstrated an association after adjustment for other parameters, including baseline SDAI. High titers of anti-CarbV IgM were associated with a poor response to MTX monotherapy, whereas a nonsignificant trend toward a better response with baricitinib and baricitinib plus MTX was observed. Other studies have shown that seropositive patients with RA have a better response to treatment modalities than seronegative patients [33] and that autoantibodies of the IgA subclass are predictive of a response to rituximab treatment [34]. However, patients with poor prognostic factors may still show a good response to treatment, as demonstrated in studies of patients positive for ACPA who responded to treatment with rituximab or abatacept [33, 35].

In addition to an association with a better clinical response, high titers of anti-CarbV IgA were associated with a greater probability of radiographic progression, as measured by change in mTSS from baseline. This finding is in agreement with those of previous studies showing that anti-CarbP are an independent risk factor for radiographic progression [36]. Taken together, these results suggest that high titers of anti-CarbV IgA are an unfavorable prognostic factor for radiographic progression. Similar to the results seen with anti-MCV antibodies and treatment response, there was no association between anti-MCV antibodies and radiographic progression.

To the best of our knowledge, no other studies have looked at the pathophysiological association between high titers of carbamylated autoantibodies and the response to specific treatments; rather, studies to date have investigated the association between antibodies and RA disease activity. This lack of similar literature prevents us from comparing and contrasting our results with those of other studies. It would be interesting to know whether the same observations can be made for other JAK inhibitors, such as tofacitinib, and this is a potential research topic for future studies. The association between high anti-CarbV IgM titers and poor response to MTX was a new and unexpected observation; if further studies confirm this result, anti-CarbV IgM titers could potentially be used as a prognostic biomarker to identify which patients with RA might not benefit from early treatment with MTX.

A limitation of this analysis is that the numbers of patients negative for the antibodies of interest in the analysis population were low, which prevented meaningful analysis of seropositive versus seronegative patients.

## Conclusion

Our results suggest that high titers of anti-CarbV IgA and IgG antibodies could represent a useful prognostic biomarker for identifying the likelihood of both the response to treatment and the potential for structural damage progression in patients with RA. Anti-MCV antibodies were not associated with either endpoint in this patient population. Further research is needed to confirm these observations.

## Supplementary information

**Additional file 1 **: **Supplementary materials**. Includes additional methodology, data relating to the association of baseline antibodies with DAS28-hsCRP, and data relating to treatment differences in the change from baseline in SDAI and SDAI response by geographic region.

## Data Availability

All data generated or analyzed during this study are included in this published article and its additional file.

## Abbreviations

ACPA: Anti-citrullinated protein antibody
BMI: Body mass index
CarbP: Carbamylated proteins
CarbV: Carbamylated vimentin
CDAI: Clinical Disease Activity Index
CFB: Change from baseline
DAS28-hsCRP: Disease Activity Score for 28-joint count with serum high-sensitivity C-reactive protein
DMARD: Disease-modifying antirheumatic drug
HAQ-DI: Health Assessment Questionnaire-Disability Index
hsCRP: High-sensitivity C-reactive protein
Ig: Immunoglobulin
JAK: Janus kinase
LRT: Likelihood-ratio chi-squared tests
MCV: Mutated citrullinated vimentin
mITT: Modified intent to treat
mLOCF: Modified last observation carried forward
MLR: Multivariable logistic regression
MMRM: Mixed-effect models for repeated measures
mTSS: Modified Total Sharp Score
MTX: Methotrexate
OR: Odds ratio
RA: Rheumatoid arthritis
RF: Rheumatoid factor
SDAI: Simplified Disease Activity Index
SDC: Smallest detectable change
STAT: Signal transducer and activator of transcription
Tyk2: Tyrosine kinase 2
