# Supplementary material for: Assessment of the association of baseline anti-CarbV and anti-MCV antibodies with response to treatment and radiographic progression in an RA population treated with either methotrexate or baricitinib: post-hoc analyses from RA-BEGIN
Source: Arthritis Res Ther. 2020 Aug 18;22:193. doi: 10.1186/s13075-020-02284-y (PMC7437063; doi:10.1186/s13075-020-02284-y)
Supplement: Supplementary file 1 — Additional file 1 : Supplementary materials. Includes additional methodology, data relating to the association of baseline antibodies with DAS28-hsCRP, and data relating to treatment differences in the change from baseline in SDAI and SDAI response by geographic region. [file 13075_2020_2284_MOESM1_ESM.docx]

# Supplementary materials

# Methods

## Assessment of radiographs for structural joint damage in RA-BEGIN

Structural joint damage was measured in RA-BEGIN using the van der Heijde-modified Total Sharp Score (mTSS) as evidenced in radiographs of the hands and feet performed at or within 4 weeks prior to baseline and at weeks 12, 24, and 52, or at the last visit in the event of early study termination. Radiographs were scored centrally and independently by two readers who were blinded to the chronological order of the radiographs, patient identifiers, and treatment groups, with adjudication by a third reader if there was disagreement beyond a predefined level.

## Statistical methods

### Handling post-baseline SDAI/DAS28-hsCRP data after the occurrence of intercurrent events

In the multivariable mixed-effect models for repeated measures (MMRM) analysis, modified last observation carried forward (mLOCF) was used to handle Simplified Disease Activity Index (SDAI) and Disease Activity Score for 28-joint count with serum high-sensitivity C-reactive protein (DAS28-hsCRP) data after the occurrence of intercurrent events, defined as events occurring after randomization and treatment initiation that either precluded observation of the variable or affected its interpretation. For patients who received rescue therapy starting from week 24, the last non-missing observation at or before rescue was carried forward to subsequent time points for evaluation. For all other patients who discontinued for any reason, the last non-missing post-baseline observation before discontinuation was carried forward to subsequent time points for evaluation. After mLOCF imputation, data from patients with non-missing observations were included in the MMRM. In the multivariable logistic regression (MLR) analysis, mTSS data at week 52 were imputed using linear extrapolation for patients who discontinued the study before week 52, had missing data, or received rescue therapy at week 24 (or any time thereafter). Linear extrapolation used baseline data and the most recent radiographic data before discontinuation, the missed radiograph, or initiation of rescue therapy. Patients with missing X-ray data or missing data for the covariates in the MLR models were excluded from analysis.

At the time of study database lock, any patients with unresolved interruptions of study drug (that is, not yet re-started, to be confirmed as a temporary interruption, and not yet permanently discontinued) were treated as having permanently discontinued study drug for the purposes of data imputation. If a patient did not have a non-missing observed record (or one imputed by other means) for a post-baseline visit, the last post-baseline measure recorded prior to the missed visit was used for this post-baseline visit.

### Natural cubic splines

A cubic spline is a mathematical function constructed of piecewise third-order polynomials, which pass through a set of m control points (knots). Specifying 3 degrees of freedom implies two interior knots plus two additional boundary knots. The polynomials are constrained so that they join smoothly at the region boundaries or knots (continuous first and second derivatives at the knots). Splines are susceptible to high variance beyond the boundary knots, where there may be fewer observations. Therefore, an additional constraint is applied to restrict these highly variable outer knots to being linear; this is termed a natural spline.

# Results

## Association of baseline antibodies with overall DAS28-hsCRP response

Patients with higher baseline anti-mutated citrullinated vimentin (MCV) (immunoglobulin [Ig]M) showed greater improvement with respect to overall DAS28-hsCRP, but this association was not statistically significant (p = 0.117) (Table S1, Fig. S1). A statistically significant treatment-by-anti-carbamylated vimentin (CarbV) [IgM] interaction was also found for DAS28-hsCRP response. Similar to the results presented for SDAI response (Fig. S2), patients who received methotrexate (MTX) at baseline who had lower baseline anti-CarbV (IgM) were more likely to show a statistically significant improvement in DAS28-hsCRP (p = 0.006). An opposite association was observed for patients treated with baricitinib who had higher baseline anti-CarbV (IgM); these patients showed a greater overall DAS28-hsCRP improvement, as was found for SDAI. The association described for patients receiving baricitinib plus MTX was also statistically significant (p = 0.042), indicating an overall improvement in DAS28-hsCRP for patients with higher baseline anti-CarbV (IgM). Despite the significant interaction between anti-CarbV (IgM) and SDAI treatment response in patients who received MTX (Figure 1C in the main manuscript), the improvement in DAS28-hsCRP response was consistently greater and statistically different for patients receiving baricitinib plus MTX or baricitinib alone compared with patients receiving MTX (Table S1 and Fig. S1).

# Tables

**Table S1** P-values for factors included in the six different MMRM used to model the association of anti-CarbV and anti-MCV antibodies with overall DAS28-hsCRP response.

| **MMRM variable** | **Anti-CarbV isotypes** | | | **Anti-MCV isotypes** | | |
| --- | --- | --- | --- | --- | --- | --- |
|  | **IgA** | **IgG** | **IgM** | **IgA** | **IgG** | **IgM** |
| Time | < 0.001 | < 0.001 | < 0.001 | < 0.001 | < 0.001 | < 0.001 |
| Treatment | < 0.001 | < 0.001 | < 0.001 | < 0.001 | < 0.001 | < 0.001 |
| Baseline antibody (only in MMRM with linear association) | NA | 0.038 | 0.809 | NA | NA | NA |
| Baseline antibody NCS (in MMRM with nonlinear association) | 0.005 | NA | NA | 0.269 | 0.250 | 0.117 |
| Baseline DAS28-hsCRP | < 0.001 | < 0.001 | < 0.001 | < 0.001 | < 0.001 | < 0.001 |
| Baseline ACPA | 0.759 | 0.800 | 0.954 | 0.844 | 0.925 | 0.678 |
| Baseline RF | 0.327 | 0.346 | 0.768 | 0.348 | 0.427 | 0.437 |
| Erosions | 0.282 | 0.415 | 0.343 | 0.293 | 0.286 | 0.281 |
| Age | 0.690 | 0.663 | 0.726 | 0.627 | 0.629 | 0.419 |
| Sex | 0.507 | 0.443 | 0.554 | 0.472 | 0.433 | 0.329 |
| BMI | 0.211 | 0.269 | 0.550 | 0.244 | 0.280 | 0.239 |
| Geographic location | 0.148 | 0.225 | 0.192 | 0.231 | 0.196 | 0.248 |
| Time-by-treatment interaction | 0.899 | 0.899 | 0.899 | 0.899 | 0.899 | 0.899 |
| Baseline antibody-by-treatment interaction | NA | NA | < 0.001 | NA | NA | NA |

Different nested MMRM were compared employing a chi-squared LRT to select between linear (baseline antibody in the MMRM) or nonlinear associations defined with an NCS (baseline antibody using an NCS in the MMRM) and to test baseline antibody-by-treatment interactions. P-values < 0.05 were considered statistically significant. The reported p-values obtained from the multivariable model are for the association between the corresponding factor and overall SDAI response, measured as CFB while controlling for the influence of all other factors included in the model: SDAI; time (visit: weeks 4, 12, 16, 20, 24, 32, 40, and 52); treatment (MTX, baricitinib 4 mg, baricitinib 4 mg + MTX); baseline antibody, in MMRM with linear association or baseline antibody using NCS, in MMRM with natural cubic splines with 3 degrees of freedom (nonlinear association); ACPA (yes/no); RF (yes/no); erosions: presence of erosions at baseline (yes/no); geographic region (Central and South America and Mexico; Europe; Japan; Rest of the World, USA and Canada); BMI; baseline antibody-by-treatment interaction, only in MMRM with (significant) baseline antibody-by-treatment interaction.

ACPA, anti-citrullinated protein antibody; BMI, body mass index; CarbV, carbamylated vimentin; CFB, change from baseline; DAS28-hsCRP, Disease Activity Score for 28-joint count with serum high-sensitivity C-reactive protein; Ig, immunoglobulin; LRT, likelihood ratio test; MCV, mutated citrullinated vimentin; MMRM, mixed model for repeated measures; MTX, methotrexate; NA, factor not estimated in the MMRM used for that specific antibody; NCS, natural cubic spline; RF, rheumatoid factor; SDAI, Simplified Disease Activity Index.

**Table S2** Multivariable logistic regression to estimate the association between anti-CarbV (IgG) and structural damage progression.

| **Logistic regression variable** | **OR** | **Lower CI** | **Upper CI** | **p-Value** |
| --- | --- | --- | --- | --- |
| Baricitinib vs. MTX | 0.530 | 0.285 | 0.963 | 0.040 |
| Baricitinib + MTX vs. MTX | 0.312 | 0.162 | 0.579 | <0.001 |
| **Baseline anti-CarbV (IgG)** | **1.000** | **0.999** | **1.002** | **0.665** |
| Baseline ACPA | 1.000 | 0.999 | 1.000 | 0.266 |
| Baseline RF | 1.000 | 0.999 | 1.001 | 0.594 |
| Erosions at baseline (yes vs. no) | 1.545 | 0.884 | 2.777 | 0.135 |
| Baseline hemoglobin | 0.809 | 0.636 | 1.023 | 0.079 |
| Baseline hsCRP | 1.017 | 1.007 | 1.026 | <0.001 |
| Age | 1.000 | 0.981 | 1.020 | 0.980 |
| Sex (male vs. female) | 0.615 | 0.283 | 1.283 | 0.205 |
| Baseline BMI | 0.945 | 0.894 | 0.996 | 0.042 |
| Smoker (yes vs. no) | 2.047 | 1.084 | 3.833 | 0.026 |
| Baseline HAQ-DI | 0.628 | 0.400 | 0.981 | 0.042 |
| Baseline CDAI | 1.023 | 1.000 | 1.047 | 0.054 |
| Europe vs. CSAM | 1.433 | 0.595 | 3.342 | 0.411 |
| Japan vs. CSAM | 1.160 | 0.497 | 2.701 | 0.731 |
| Rest of World vs. CSAM | 1.089 | 0.476 | 2.446 | 0.838 |
| USA and Canada vs. CSAM | 0.642 | 0.263 | 1.471 | 0.309 |

Adjusted ORs from the MLR model with corresponding 95% CIs and p-values. P-values < 0.05 were considered statistically significant.

ACPA, anti-citrullinated protein antibody; BMI, body mass index; CarbV, carbamylated vimentin; CDAI, Clinical Disease Activity Index; CI, confidence interval; CSAM, Central and South America; HAQ-DI, Health Assessment Questionnaire-Disability Index; hsCRP, high-sensitivity C-reactive protein; Ig, immunoglobulin; MLR, multivariable logistic regression; MTX, methotrexate; OR, odds ratio; RF, rheumatoid factor. Bold formatting indicates the association between the antibody isotype and structural damage progression.

**Table S3** Multivariable logistic regression to estimate the association between anti-CarbV (IgM) and structural damage progression.

| **Logistic regression variable** | **OR** | **Lower CI** | **Upper CI** | **p-Value** |
| --- | --- | --- | --- | --- |
| Baricitinib vs. MTX | 0.532 | 0.287 | 0.965 | 0.041 |
| Baricitinib + MTX vs. MTX | 0.307 | 0.159 | 0.573 | <0.001 |
| **Baseline anti-CarbV (IgM)** | **1.001** | **0.999** | **1.003** | **0.336** |
| Baseline ACPA | 1.000 | 0.999 | 1.000 | 0.252 |
| Baseline RF | 1.000 | 0.999 | 1.001 | 0.928 |
| Erosions at baseline (yes vs. no) | 1.546 | 0.888 | 2.769 | 0.132 |
| Baseline hemoglobin | 0.807 | 0.635 | 1.021 | 0.076 |
| Baseline hsCRP | 1.017 | 1.008 | 1.026 | <0.001 |
| Age | 1.001 | 0.982 | 1.020 | 0.941 |
| Sex (male vs. female) | 0.603 | 0.277 | 1.262 | 0.189 |
| Baseline BMI | 0.945 | 0.894 | 0.996 | 0.039 |
| Smoker (yes vs. no) | 2.004 | 1.059 | 3.760 | 0.031 |
| Baseline HAQ-DI | 0.618 | 0.392 | 0.966 | 0.618 |
| Baseline CDAI | 1.023 | 1.000 | 1.047 | 1.023 |
| Europe vs. CSAM | 1.513 | 0.623 | 3.567 | 1.513 |
| Japan vs. CSAM | 1.161 | 0.500 | 2.693 | 1.161 |
| Rest of World vs. CSAM | 1.111 | 0.487 | 2.488 | 0.799 |
| USA and Canada vs. CSAM | 0.678 | 0.277 | 1.565 | 0.376 |

Adjusted ORs from the MLR model with corresponding 95% CIs and p-values. P-values < 0.05 were considered statistically significant.

ACPA, anti-citrullinated protein antibody; BMI, body mass index; CarbV, carbamylated vimentin; CDAI, Clinical Disease Activity Index; CI, confidence interval; CSAM, Central and South America; HAQ-DI, Health Assessment Questionnaire-Disability Index; hsCRP, high-sensitivity C-reactive protein; Ig, immunoglobulin; MLR, multivariable logistic regression; MTX, methotrexate; OR, odds ratio; RF, rheumatoid factor. Bold formatting indicates the association between the antibody isotype and structural damage progression.

**Table S4** Multivariable logistic regression to estimate the association between anti-MCV (IgA) and structural damage progression.

| **Logistic regression variable** | **OR** | **Lower CI** | **Upper CI** | **p-Value** |
| --- | --- | --- | --- | --- |
| Baricitinib vs. MTX | 0.532 | 0.286 | 0.967 | 0.041 |
| Baricitinib + MTX vs. MTX | 0.313 | 0.162 | 0.581 | <0.001 |
| **Baseline anti-MCV (IgA)** | **1.000** | **0.996** | **1.002** | **0.879** |
| Baseline ACPA | 1.000 | 1.000 | 1.000 | 0.338 |
| Baseline RF | 1.000 | 0.999 | 1.001 | 0.560 |
| Erosions at baseline (yes vs. no) | 1.524 | 0.876 | 2.728 | 0.144 |
| Baseline hemoglobin | 0.808 | 0.636 | 1.023 | 0.079 |
| Baseline hsCRP | 1.017 | 1.008 | 1.026 | <0.001 |
| Age | 1.000 | 0.981 | 1.020 | 0.985 |
| Sex (male vs. female) | 0.615 | 0.283 | 1.284 | 0.206 |
| Baseline BMI | 0.945 | 0.893 | 0.995 | 0.038 |
| Smoker (yes vs. no) | 2.061 | 1.091 | 3.860 | 0.024 |
| Baseline HAQ-DI | 0.631 | 0.401 | 0.985 | 0.043 |
| Baseline CDAI | 1.024 | 0.999 | 1.048 | 0.049 |
| Europe vs. CSAM | 1.431 | 0.591 | 3.353 | 0.415 |
| Japan vs. CSAM | 1.178 | 0.507 | 2.737 | 0.703 |
| Rest of World vs. CSAM | 1.073 | 0.465 | 2.425 | 0.867 |
| USA and Canada vs. CSAM | 0.649 | 0.266 | 1.485 | 0.320 |

Adjusted ORs from the MLR model with corresponding 95% CIs and p-values. P-values < 0.05 were considered statistically significant.

ACPA, anti-citrullinated protein antibody; BMI, body mass index; CDAI, Clinical Disease Activity Index; CI, confidence interval; CSAM, Central and South America; HAQ-DI, Health Assessment Questionnaire-Disability Index; hsCRP, high-sensitivity C-reactive protein; Ig, immunoglobulin; MCV, mutated citrullinated vimentin; MLR, multivariable logistic regression; MTX, methotrexate; OR, odds ratio; RF, rheumatoid factor. Bold formatting indicates the association between the antibody isotype and structural damage progression.

**Table S5** Multivariable logistic regression to estimate the association between anti-MCV (IgG) and structural damage progression.

| **Logistic regression variable** | **OR** | **Lower CI** | **Upper CI** | **p-Value** |
| --- | --- | --- | --- | --- |
| Baricitinib vs. MTX | 0.517 | 0.277 | 0.941 | 0.034 |
| Baricitinib + MTX vs. MTX | 0.320 | 0.166 | 0.597 | <0.001 |
| **Baseline anti-MCV (IgG)** | **1.001** | **1.000** | **1.001** | **0.116** |
| Baseline ACPA | 1.000 | 0.999 | 1.000 | 0.109 |
| Baseline RF | 1.000 | 0.999 | 1.001 | 0.704 |
| Erosions at baseline (yes vs. no) | 1.545 | 0.887 | 2.769 | 0.132 |
| Baseline hemoglobin | 0.806 | 0.634 | 1.019 | 0.074 |
| Baseline hsCRP | 1.017 | 1.008 | 1.026 | <0.001 |
| Age | 1.001 | 0.982 | 1.020 | 0.918 |
| Sex (male vs. female) | 0.606 | 0.279 | 1.269 | 0.194 |
| Baseline BMI | 0.945 | 0.894 | 0.996 | 0.042 |
| Smoker (yes vs. no) | 2.023 | 1.070 | 3.794 | 0.028 |
| Baseline HAQ-DI | 0.617 | 0.392 | 0.963 | 0.034 |
| Baseline CDAI | 1.023 | 1.000 | 1.047 | 0.053 |
| Europe vs. CSAM | 1.608 | 0.659 | 3.807 | 0.286 |
| Japan vs. CSAM | 1.167 | 0.501 | 2.716 | 0.719 |
| Rest of World vs. CSAM | 1.223 | 0.528 | 2.788 | 0.634 |
| USA and Canada vs. CSAM | 0.596 | 0.242 | 1.376 | 0.239 |

Adjusted ORs from the MLR model with corresponding 95% CIs and p-values. P-values < 0.05 were considered statistically significant.

ACPA, anti-citrullinated protein antibody; BMI, body mass index; CDAI, Clinical Disease Activity Index; CI, confidence interval; CSAM, Central and South America; HAQ-DI, Health Assessment Questionnaire-Disability Index; hsCRP, high-sensitivity C-reactive protein; Ig, immunoglobulin; MCV, mutated citrullinated vimentin; MLR, multivariable logistic regression; MTX, methotrexate; OR, odds ratio; RF, rheumatoid factor. Bold formatting indicates the association between the antibody isotype and structural damage progression.

**Table S6** Multivariable logistic regression to estimate the association between anti-MCV (IgM) and structural damage progression.

| **Logistic regression variable** | **OR** | **Lower CI** | **Upper CI** | **P-value** |
| --- | --- | --- | --- | --- |
| Baricitinib vs. MTX | 0.532 | 0.286 | 0.967 | 0.042 |
| Baricitinib + MTX vs. MTX | 0.313 | 0.162 | 0.582 | <0.001 |
| **Baseline anti-MCV (IgM)** | **1.002** | **0.999** | **1.006** | **0.207** |
| Baseline ACPA | 1.000 | 0.999 | 1.000 | 0.148 |
| Baseline RF | 1.000 | 0.999 | 1.001 | 0.974 |
| Erosions at baseline (yes vs. no) | 1.568 | 0.899 | 2.812 | 0.121 |
| Baseline hemoglobin | 0.803 | 0.632 | 1.016 | 0.069 |
| Baseline hsCRP | 1.017 | 1.008 | 1.026 | <0.001 |
| Age | 1.000 | 0.981 | 1.019 | 0.998 |
| Sex (male vs. female) | 0.594 | 0.272 | 1.247 | 0.178 |
| Baseline BMI | 0.945 | 0.894 | 0.996 | 0.041 |
| Smoker (yes vs. no) | 2.002 | 1.059 | 3.749 | 0.031 |
| Baseline HAQ-DI | 0.625 | 0.398 | 0.976 | 0.040 |
| Baseline CDAI | 1.023 | 1.000 | 1.047 | 0.057 |
| Europe vs. CSAM | 1.442 | 0.599 | 3.363 | 0.403 |
| Japan vs. CSAM | 1.187 | 0.511 | 2.759 | 0.689 |
| Rest of World vs. CSAM | 1.058 | 0.467 | 2.356 | 0.890 |
| USA and Canada vs. CSAM | 0.638 | 0.261 | 1.467 | 0.304 |

Adjusted ORs from the MLR model with corresponding 95% CIs and p-values. P-values < 0.05 were considered statistically significant.

ACPA, anti-citrullinated protein antibody; BMI, body mass index; CDAI, Clinical Disease Activity Index; CI, confidence interval; CSAM, Central and South America; HAQ-DI, Health Assessment Questionnaire-Disability Index; hsCRP, high-sensitivity C-reactive protein; Ig, immunoglobulin; MCV, mutated citrullinated vimentin; MLR, multivariable logistic regression; MTX, methotrexate; OR, odds ratio; RF, rheumatoid factor. Bold formatting indicates the association between the antibody isotype and structural damage progression.

# Figures

**Fig. S1** Adjusted means for the association of baseline anti-CarbV and anti-MCV antibodies with overall change from baseline in overall DAS28-hsCRP response. A) anti-CarbV (IgA); B) anti-CarbV (IgG); C) anti-CarbV (IgM); D) anti-MCV (IgA); E) anti-MCV (IgG); F) anti-MCV (IgM).

Adjusted overall DAS28-hsCRP means as a function of serum baseline antibody concentrations were estimated using multivariable MMRMs with continuous covariates fixed at their mean values and categorical covariates fixed at their proportional distribution in the data. Overall DAS28-hsCRP response was estimated from the MMRM, averaging SDAI responses at all post-baseline visits (weeks 4, 12, 16, 20, 24, 32, 40, and 52). A) A statistically significant nonlinear association was found for anti-CarbV (IgA). Patients with higher baseline anti-CarbV (IgA) were more likely to show an improved DAS28-hsCRP response (p = 0.005). B) A significant linear association was found for anti-CarbV (IgG) (p = 0.038). C) A significant baseline anti-CarbV (IgM)-by-treatment interaction was found (p < 0.001). The association between anti-CarbV (IgM) and DAS28-hsCRP depended on the treatment received at baseline. Patients randomized to MTX who had higher baseline anti-CarbV (IgM) were more likely to show less improvement in DAS28-hsCRP (p = 0.006). An opposite association was observed for patients randomized to baricitinib. Patients randomized to baricitinib who had higher baseline anti-CarbV (IgM) showed greater improvement in overall DAS28-hsCRP (baricitinib, p = 0.0905; baricitinib + MTX, p = 0.0422). D) No statistically significant association was observed for anti-MCV (IgA) (p = 0.269). E) No statistically significant association was observed for anti-MCV (IgG) (p = 0.250). F) Patients with higher baseline anti-MCV (IgM) showed greater improvement in overall DAS28-hsCRP, but the association was not statistically significant (p = 0.117). BARI, baricitinib; CarbV, carbamylated vimentin; DAS28-hsCRP, Disease Activity Score for 28-joint count with serum high-sensitivity C-reactive protein; Ig, immunoglobulin; MCV, mutated citrullinated vimentin; mLOCF, modified last observation carried forward; MMRM, mixed model for repeated measures; MTX, methotrexate; SDAI Simplified Disease Activity Index.

**Fig. S2** Adjusted treatment differences for change from baseline in SDAI at each study visit, estimated using MMRM to model baseline anti-CarbV (IgA).

Adjusted means and 95% CI for treatment differences with respect to change from baseline in SDAI were estimated using multivariable MMRMs with continuous covariates fixed at their mean values and categorical covariates fixed at their proportional distribution in the data. Overall SDAI was estimated from the MMRM, averaging all post-baseline visits (weeks 4, 12, 16, 20, 24, 32, 40, and 52). Between-group differences for baricitinib vs. MTX were as follows: overall, -6.594, p < 0.0001; week 4, -7.764, p < 0.0001; week 12, -6.852, p < 0.0001; week 16, -7.728, p < 0.0001; week 20, -5.644, p < 0.0001; week 24, -5.740, p < 0.0001; week 32, -6.349, p < 0.0001; week40, -6.000, p < 0.0001; week 52, -6.675, p < 0.0001. The between-group differences for baricitinib + MTX vs. MTX were as follows: overall, -8.591, p < 0.0001; week 4, -9.583, p < 0.0001; week 12, -9.181, p < 0.0001; week 16, -9.519, p < 0.0001; week 20, -8.372, p < 0.0001; week 24, -7.685, p < 0.0001; week 32, -8.185, p < 0.0001; week40, -7.637, p < 0.0001; week 52, -8.569, p < 0.0001. BARI, baricitinib; CarbV, carbamylated vimentin; CI, confidence interval; Ig, immunoglobulin; mLOCF, modified last observation carried forward; MMRM, mixed model for repeated measures; MTX, methotrexate; SDAI, Simplified Disease Activity Index.

**Fig. S3** Adjusted overall SDAI response by geographic region.

Adjusted overall means and 95% CI for differences between geographic locations with respect to change from baseline in SDAI were estimated using multivariable MMRMs with continuous covariates fixed at their mean values and categorical covariates fixed at their proportional distribution in the data. Overall SDAI was estimated from MMRM, averaging all post-baseline visits (weeks 4, 12, 16, 20, 24, 32, 40, and 52). When other regions were compared with CSAM, between-group differences were as follows: Rest of World, 1.320, p = 0.366; Europe, 2.251, p = 0.166; Japan, 2.998, p = 0.058; and USA and Canada, 4.538, p = 0.001. When other regions were compared with the USA and Canada, between-group differences were as follows: CSAM, -4.538, p = 0.001; Europe, -2.287, p = 0.176; Japan, -1.541, p=0.361; Rest of World, -3.218, p = 0.045. CI, confidence interval; CSAM, Central and South America; mLOCF, modified last observation carried forward; MMRM, mixed model for repeated measures; SDAI, Simplified Disease Activity Index.
